# Specific Inhibition of the Nuclear Exporter Exportin-1 Attenuates Kidney Cancer Growth

**DOI:** 10.1371/journal.pone.0113867

**Published:** 2014-12-02

**Authors:** Hiromi I. Wettersten, Yosef Landesman, Sharon Friedlander, Sharon Shacham, Michael Kauffman, Robert H. Weiss

**Affiliations:** 1 Division of Nephrology, Dept. of Internal Medicine, University of California Davis, Davis, California, United States of America; 2 Cancer Center, University of California Davis, Davis, California, United States of America; 3 Karyopharm Therapeutics Inc., Natick, Massachusetts, United States of America; 4 Medical Service, Sacramento VA Medical Center, Sacramento, California, United States of America; Roswell Park Cancer Institute, United States of America

## Abstract

**Purpose:**

Despite the advent of FDA-approved therapeutics to a limited number of available targets (kinases and mTOR), PFS of kidney cancer (RCC) has been extended only one to two years due to the development of drug resistance. Here, we evaluate a novel therapeutic for RCC which targets the exportin-1 (XPO1) inhibitor.

**Materials and Methods:**

RCC cells were treated with the orally available XPO1 inhibitor, KPT-330, and cell viability and Annexin V (apoptosis) assays, and cell cycle analyses were performed to evaluate the efficacy of KPT-330 in two RCC cell lines. Immunoblotting and immunofluorescence analysis were performed to validate mechanisms of XPO1 inhibition. The efficacy and on-target effects of KPT-330 were further analyzed in vivo in RCC xenograft mice, and KPT-330-resistant cells were established to evaluate potential mechanisms of KPT-330 resistance.

**Results:**

KPT-330 attenuated RCC viability through growth inhibition and apoptosis induction both in vitro and in vivo, a process in which increased nuclear localization of p21 by XPO1 inhibition played a major role. In addition, KPT-330 resistant cells remained sensitive to the currently approved for RCC multi-kinase inhibitors (sunitinib, sorafenib) and mTOR inhibitors (everolimus, temsirolimus), suggesting that these targeted therapeutics would remain useful as second line therapeutics following KPT-330 treatment.

**Conclusion:**

The orally-available XPO1 inhibitor, KPT-330, represents a novel target for RCC whose in vivo efficacy approaches that of sunitinib. In addition, cells resistant to KPT-330 retain their ability to respond to available RCC therapeutics suggesting a novel approach for treatment in KPT-330-naïve as well as -resistant RCC patients.

## Introduction

Kidney cancer (renal cell carcinoma; RCC) is the 13^th^ most common cancer worldwide and is one of few cancers whose incidence is increasing, a finding not merely due to improved diagnostic techniques [1], [2]. The signs and symptoms of RCC are frequently subtle or even absent, such that more than half of patients are diagnosed incidentally, and often at the metastatic stage, while being evaluated for other diseases such as acute kidney injury [3]. While the five-year survival for those who present with localized RCC is more than 70%, for those with metastatic disease, the five-year survival drops to a dismal 16 to 32%. Approximately half of RCC patients develop advanced disease and require systemic therapy. Despite the advent of several FDA-approved targeted therapeutics over the past several years, which are limited to multi-kinase inhibitors and mTOR inhibitors, progression free survival (PFS) is extended only one to two years with these targeted therapeutics due largely to the development of drug resistance [4]. Thus, it is critical to develop novel therapeutics to targets other than kinases and the mTOR pathway.

p21 was originally described as a cyclin-dependent-kinase inhibitor (CKI) of cyclin-CDK2, -CDK1, and -CDK4/6 complexes whose expression is classically regulated by p53 [5]. However, over the years it has been shown to have pleiotropic, and at times seemingly contradictory, effects on cell proliferation, apoptosis, and senescence in cancer and in vascular disease independent from p53 [5], [6]. In general, when p21 is localized within the nucleus, it binds to cyclin-CDK complexes thereby inhibiting their function in cell cycle progression, resulting in cell cycle arrest [5]. However, when p21 is localized within the cytosolic compartment, it inhibits apoptosis by complexing with pro-apoptotic proteins such as pro-caspase-3 or ASK [7], [8]. Consistent with these putative mechanisms, previous work in our laboratory has demonstrated that increased cytosolic p21 is an indicator of poor prognosis in RCC patients [9], a finding also observed in other cancers [10], [11].

The nuclear exporter, exportin11 (XPO1; CRM1), controls the nucleo-cytoplasmic localization of more than 200 Nuclear Export Signal (NES)-containing proteins, many of which are tumor suppressor proteins (TSPs), including p21 [12]. It has been previously shown that XPO1 inhibitors have a therapeutic effect in RCC [13]. In this study, we tested efficacy of KPT-330, the orally available XPO1 inhibitor which is currently in phase I/II clinical trials, to evaluate its potential clinical, either singly or as combination treatment, in advanced RCC. We now show that, likely through a mechanism related to subcellular localization of p21, this orally-available XPO1 inhibitor represents a viable new therapeutic acting on a heretofore untested target in RCC.

## Materials and Methods

### Cell culture

The RCC cell lines ACHN and 786-O were obtained from the American Type Culture Collection (Rockville, MD) and regularly evaluated for the presence of Mycoplasma. Normal human kidney proximal epithelial (NHK) cells were obtained from Lonza (Allendale, NJ). All cells were maintained in Dulbecco’s modified Eagle’s medium supplemented with 10% fetal bovine serum (FBS), 100 units/mL streptomycin, and 100 mg/mL penicillin at 5% CO_2_ at 37°C. These two cell lines were chosen to examine efficacy of KPT-330 in both primary tumor derived cells (786-O; which can be considered “early” tumors) as well as metastatic cells (ACHN).

### Materials

Lipofectamine RNAiMAX transfection reagent, Stealth RNAi negative control siRNA, and Stealth RNAi XPO1 siRNA were obtained from Life Technologies (Grand Island, NY). KPT-330 was synthesized by Karyopharm Therapeutics (Natick, MA). Sunitinib and sorafenib free base were obtained from LC Laboratories (Worburn, MA). Everolimus and temsirolimus were gifts from Dr. Prasit at Inception Sciences (San Diego, CA). Dimethyl sulfoxide (DMSO) and mouse monoclonal anti-β-actin antibody were obtained from Sigma (St. Louis, MO). Rabbit polyclonal anti-XPO1 antibody and mouse monoclonal anti-p53 antibody were obtained from Santa Cruz Biotechnology, Inc. (Santa Cruz, CA). Mouse monoclonal anti-p21WAF1/Cip antibody was obtained from Millipore (Billerica, MA). Rabbit monoclonal anti-p21WAF1/Cip antibody and anti-rabbit IgG (H+L), F(ab′)2 Fragment (Alexa Fluor 488 Conjugate) were obtained from Cell Signaling Technology, Inc. (Beverly, MA). Goat anti-mouse and goat anti-rabbit HRP conjugated IgG were obtained from Bio-Rad (Hercules, CA). VECTASHIELD HardSet Mounting Medium with DAPI was obtained from Vector Laboratories (Burlingame, CA).

KPT-330, sunitinib, sorafenib, everolimus, and temsirolimus were dissolved in DMSO for in vitro studies. KPT-330 was combined with vehicles Poloxamer 188 (Pluronic F68; obtained from Spectrum Laboratory Products, Inc.) and PVP K-29/32 (Plasdone K-29/32; obtained from ISP technologies, Inc.) in solution and sunitnib was dissolved in vegetable oil for the in vivo study.

### Immunoblotting

Immunoblotting was as described previously [14]. Briefly, after appropriate treatments, the cells were washed with phosphate buffered saline (PBS), lysed in lysis buffer, and cell lysates were immunoblotted. Membranes were blocked in 5% nonfat dry milk for one hour at room temperature and probed with appropriate antibodies. Membranes were then probed with HRP tagged anti-mouse or anti-rabbit IgG antibodies. Signal was detected using enhanced chemiluminescence (ECL) solutions. Densitometry analysis was performed utilizing the ImageJ 1.440 software (http://imagej.nih.gov/ij) and, after normalization to loading control (β-actin) is indicated above each immunoblot.

### Immunofluorescence

After indicated treatment in eight well chamber slides, immunofluorescence was performed as described previously [14]. Briefly, the cells were fixed in 4% paraformaldehyde and placed in blocking buffer. Subsequently, the cells were incubated with the indicated antibody, incubated with anti-rabbit IgG (H+L), F(ab′)_2_ Fragment (Alexa Fluor 488 Conjugate), and coverslipped with VECTASHIELD with DAPI. The specimens were examined by confocal microscopy.

### MTT assay

Cell viability assay was performed as described previously [14]. Briefly, cells were plated in 96 well plates, and after appropriate treatments, the cells were incubated in MTT solution/media mixture. Then, the MTT solution was removed and the blue crystalline precipitate in each well was dissolved in DMSO. Visible absorbance of each well at 540 nm was quantified using a microplate reader.

### Cell cycle analysis

Cell cycle analysis was performed utilizing the MUSE Cell Analyzer (Millipore, Billerica, MA) following the manufacturer’s instruction. Briefly, after the indicated treatments, the cells were washed with PBS and stained with propidium iodide (PI). After staining, the cells were processed for cell cycle analysis.

### Annexin V assay

Annexin V & Dead Cell Assay was performed utilizing the MUSE Cell Analyzer following manufacturer’s instruction. Briefly, after appropriate treatments, the cells were incubated with Annexin V and Dead Cell Reagent (7-AAD) and the events for dead, late apoptotic, early apoptotic, and live cells were counted.

### Immunohistochemistry

Biogenex I6000 automated immunostainer was used to carry out IHC on formalin fixed paraffin embedded xenografts. Antigen was retrieved by steaming with Cell Marque Declere reagent. Background was blocked with Biogenex Power block. Primary antibody was applied for 1 hour at room temperature followed by detection with the two-step, Hi-Def Polymer Detection kit from Cell Marque, followed by Cell Marque DAB chromagen. Samples were counterstained with hematoxylin, dehydrated, cleared, and coverslipped. We used the following primary antibodies: p21 (Abcam) and Ki67 (Cell Marque). The Millipore Apoptosis kit was used according to manufacturers’ protocol.

### siRNA transfection

Lipofectamine RNAiMAX transfection reagent was mixed with Stealth RNAi siRNA following the manufacturer’s instructions. Then cells were incubated with the mixture in growth media without penicillin/streptomycin for appropriate time for transfection of siRNAs and knockdown was confirmed by immunoblotting.

### ACHN xenograft mouse experiment

The UC Davis Institutional Animal Care and Use Committee (IACUC) committee specifically approved this study. All animal procedures were performed in compliance with the University of California IACUC. Male athymic Nu/Nu mice were each injected with 5×10^5^ ACHN cells subcutaneously in the flank region. Tumor progression was monitored weekly with a caliper (tumor volume = length x width x width/2). When tumor sizes reached around 80–100 mm^3^, mice were divided randomly into five groups (vehicle, sunitinib, KPT-330 low or high dose, or KPT-330 low and sunitinib) for drug treatments. For the vehicle group, vehicle solutions (Pluronic F-68 and PVP K-29/32 mixture and vegetable oil) were given orally (twice per week and five times per week respectively, n = 8). For the sunitinib group, the vehicle solution for KPT-330, Pluronic F-68 and PVP K-29/32 mixture, and sunitinib (40 mg/kg) were given orally (twice per week and five times per week respectively, n = 8) [15]. For the KPT-330 low group, low dose of KPT-330 (7.5 mg/kg) and vegetable oil was given orally (twice per week and five times per week respectively, n = 8). For the KPT-330 high group, high dose of KPT-330 (15 mg/kg) and vegetable oil was given orally (twice per week and five times per week respectively, n = 8). For the KPT-330 low and sunitinib group, low dose of KPT-330 (7.5 mg/kg) and sunitinib (40 mg/kg) were given orally (twice per week and five times per week respectively, n = 8). After 25 days of treatment, the animals were sacrificed and tumor tissues were collected in 10% formalin for immunohistochemistry analysis.

### Statistical methods

For in vitro studies, comparisons of mean values were performed using the independent samples t-test. A p-value of <0.05 was considered significant. For the in vivo study, tumor growth was compared pairwise between all treatments using the Tukey HSD method.

## Results

### Attenuation of XPO1 by KPT-330 inhibits RCC viability through cell cycle arrest and induction of apoptosis

**Figure 1:**
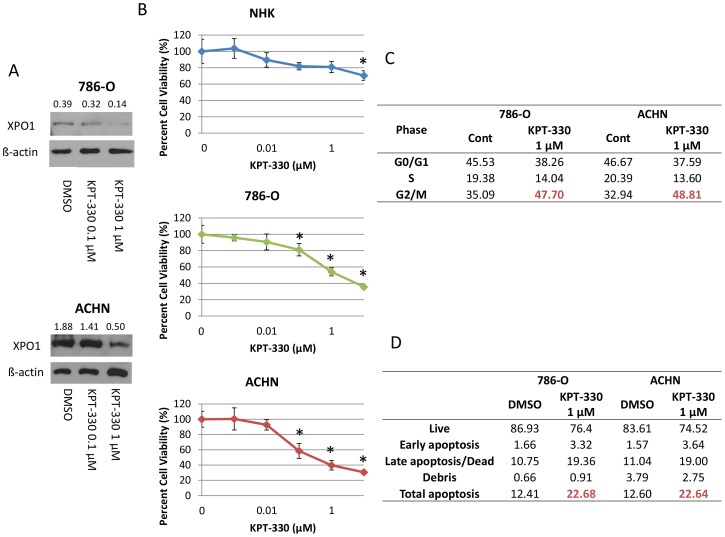
Attenuation of XPO1 by KPT-330 inhibited RCC viability through cell cycle arrest and apoptosis induction. The RCC cells 786-O and ACHN were grown to ∼60% confluence and treated with KPT-330 at indicated doses for 24 hours and immunoblotting was accomplished with specific antibodies (A). RCC and NHK cells were grown to ∼30% confluence, treated with KPT-330 at indicated doses for 72 hours, and MTT assays were performed (B). Cell cycle analysis (C) and Annexin V assay (D) were performed after 24 hour incubation with DMSO (Cont) or KPT-330 (1 µM). * P<0.05 compared to control. Error bars indicate standard deviation.

We first confirmed that KPT-330 attenuated levels of XPO1 in two RCC cell lines at a similar magnitude (from 0.1 µM) to that which was observed with previously developed inhibitors of this nuclear transporter (Fig. 1A) [13]. To evaluate efficacy of XPO1 inhibition by KPT-330 towards cell survival, we determined that cell viability in response to this inhibitor was evident at a concentration of 0.1 µM in RCC cell lines but at an order of magnitude higher dose (10 µM) in normal renal tubular epithelial (NHK) cells (Fig. 1B), a finding possibly due to increased XPO1 levels in RCC tissues as compared to normal kidney tissues which we have previously shown [13]. To begin to investigate the mechanisms of the KPT-330-induced decreased cell viability in RCC, cell cycle and apoptosis analysis were performed. When incubated with KPT-330, both RCC cell lines showed an increase of cells in the G2/M phase of the cell cycle of more than 10% (Fig. 1C), as well as an apoptotic cell fraction of approximately 10% (Fig. 1D and Fig. S1), suggesting that decreased viability of RCC cells by KPT-330 occurs via both cell cycle arrest and induction of apoptosis.

### XPO1 inhibition confined p21 to the nucleus in RCC cells but not in a normal renal epithelial cell line

**Figure 2:**
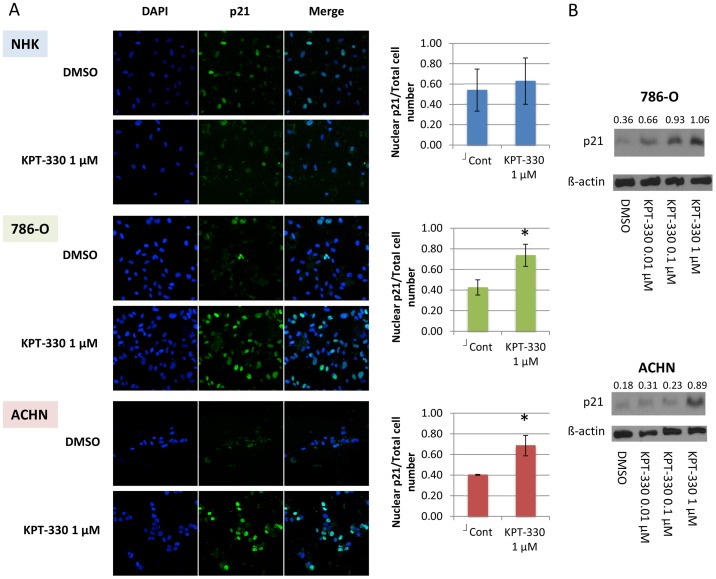
XPO1 inhibition by KPT-330 confined p21 in the nucleus in RCC cells but not in NHK cells. The RCC cells 786-O and ACHN were grown to ∼60% confluence and treated with KPT-330 at indicated doses for 24 hours. Immunofluorescence was performed by confocal microscopy (20x) and the number of cells in which p21 was predominantly in the nucleus were counted in three randomly selected fields and divided by the total cell number (A). Immunoblotting was accomplished with specific antibodies (B). Blue, nucleus (DAPI); Green, p21. *P<0.05 compared to control. Error bars indicate standard deviation.

Increased cytosolic p21, which has been shown to inhibit apoptosis [5] and thus possibly thwart tumor surveillance, is an indicator of poor prognosis for RCC [9]. For this reason, and in light of the known property of nuclear p21 in attenuating cell cycle progression, we asked whether nuclear p21 is required for KPT-330 to cause the observed effects in RCC cells. When visualized by immunofluorescence cytochemistry, p21 was shown to be largely confined to the nucleus after KPT-330 treatment in both RCC cell lines (Fig. 2A). Consistent with the cell cycle data, KPT-330 failed to alter localization of p21 in the NHK cells (Fig. 2A); this may explain the failure of KPT-330 to decrease survival in the normal cells and is relevant to future clinical translation of these findings (*vide infra*). Interestingly, p21 levels were augmented by KPT-330 in RCC cells (Fig. 2B), suggesting that p21 was not only confined in the nucleus by KPT-330 but also showed increased levels, likely mediating the observed G2/M cell cycle arrest (see Fig. 1C and [16]).

### siRNA inhibition of XPO1 increased nuclear p21 in RCC cells

**Figure 3:**
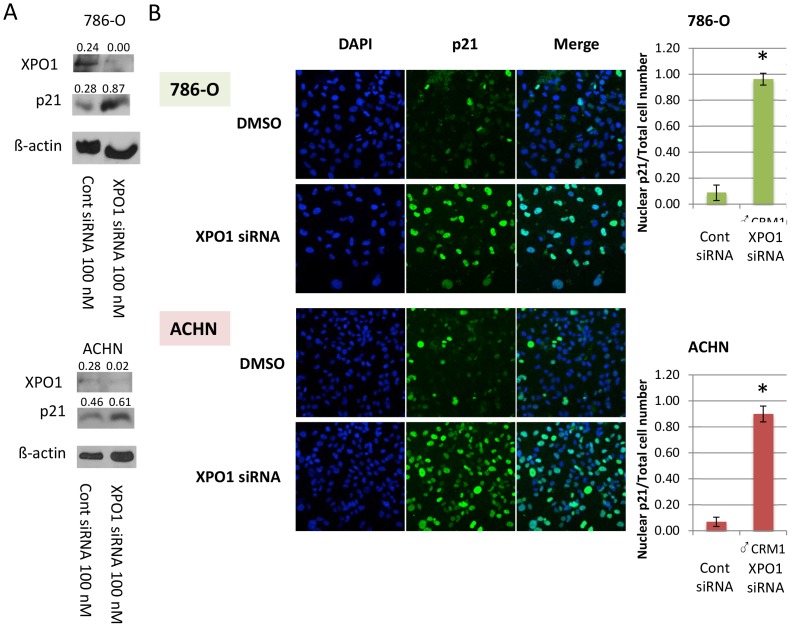
Inhibition of XPO1 by siRNA transfection increased nuclear p21 in RCC cells. The RCC (786-O and ACHN) cells were grown to ∼60% confluence and were transfected with scrambled sequence control or XPO1-specific siRNA for 48 hours. Immunoblotting was accomplished with specific antibodies (A) and immunofluorescence was performed by confocal microscopy (20x) (B). The number of cells in which p21 was predominantly in the nucleus were counted in three randomly selected fields and divided by the total cell number. Blue, nucleus (DAPI); Green, p21. *P<0.05 compared to control. Error bars indicate standard deviation.

To demonstrate specificity of KPT-330 on levels and localization of p21, and that these effects are due to XPO1 inhibition, RCC (786-O and ACHN) cells were transfected with either an siRNA specific for XPO1 or a scrambled sequence control siRNA. Both cell lines demonstrated decreased levels of XPO1 accompanied by an increase in total p21 upon XPO1 knockdown (Fig. 3A), with immunofluorescence staining showing that p21 was largely confined to the nucleus under these conditions (Fig. 3B), similar to what was observed with KPT-330 (see Fig. 2B). Thus, the effect of KPT-330 on p21 was due specifically to XPO1 attenuation.

### Orally administered KPT-330 attenuated tumor growth, associated with increased nuclear p21

**Figure 4:**
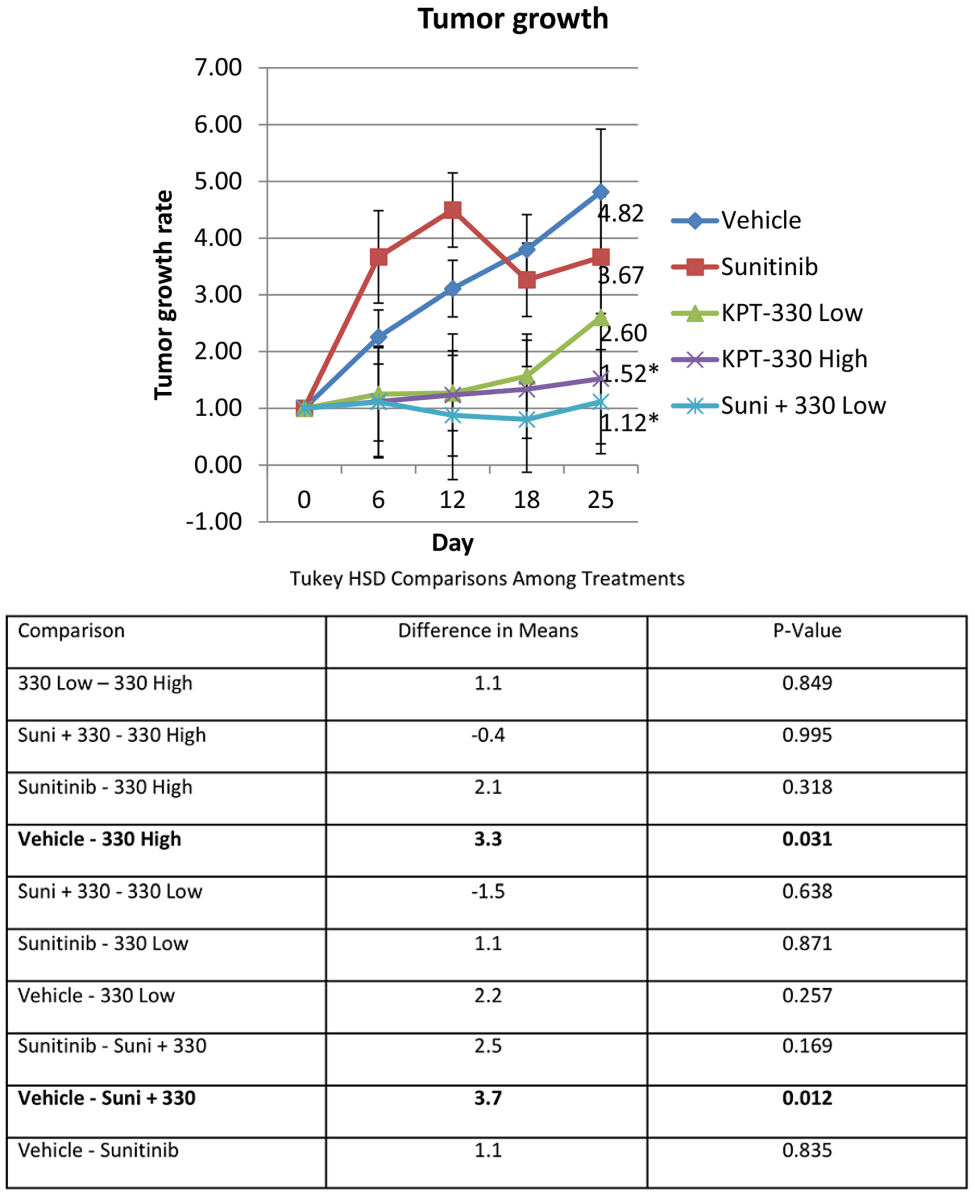
Orally administered KPT-330 attenuated tumor growth. 5×10^5^ ACHN cells were subcutaneously injected to flank region. Once the tumors were palpable, the mice were treated with vehicle, sunitinib (40 mg/kg), KPT-330 low (7.5 mg/kg), KPT-330 high (15 mg/kg) or sunitinib and KPT-330 low for 25 days as discussed in Materials and Methods. Tumor growth was monitored by caliper (tumor volume = length x width x width/2) and tumor growth rate (tumor volume at day X/tumor volume at day 0) was calculated. *P<0.05 compared to control. Error bars indicate standard error.

To begin to translate this work to the bedside, the efficacy of KPT-330 in an RCC mouse xenograft model was evaluated. Once the subcutaneously-implanted tumors became palpable, the mice were treated with sunitinib (40 mg/kg), KPT-330 low (7.5 mg/kg) and high (15 mg/kg) doses, a combination of sunitinib and KPT-330 low dose, or vehicle (which was the same for KPT-330 and sunitinib). Sunitinib was utilized both as a drug control and as a potential combination treatment partner since sunitinib is the first line therapy for RCC [17]. Both KPT-330 high dose and the combination of sunitinib and KPT-330 low dose inhibited RCC growth significantly as compared to the vehicle (Fig. 4), suggesting that KPT-330 can be utilized as single therapy and is also beneficial when combined with the current first line therapeutic, sunitinib.

**Figure 5:**
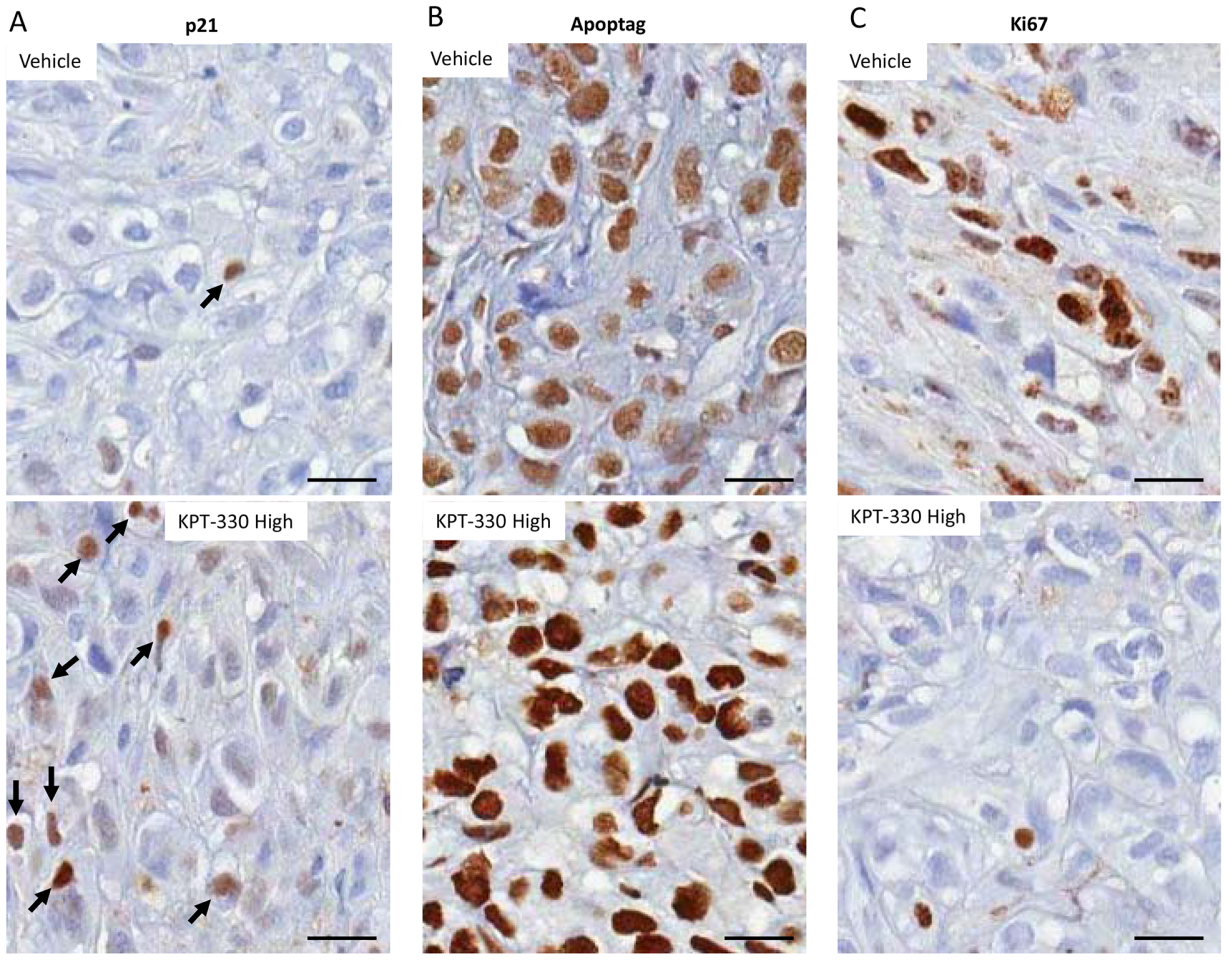
KPT-330 confined p21 in the nucleus resulting in inhibition of cell proliferation and induction of apoptosis in vivo. After the conclusion of the experiment described in Fig. 6, xenograft tissues were collected and processed for immunohistochemistry with the indicated antibodies as described in Materials and Methods. Bar = 20 µm.

To validate on-target effects of KPT-330, the xenograft tissues were processed after sacrifice for immunohistochemistry analysis. As compared to the control xenograft tissues, KPT-330 (high dose) treated animals showed an increase in p21 nuclear staining, an increase in the apoptosis marker Apoptag staining, and a decrease in staining by the proliferation marker Ki67 (Fig. 5), all consistent with our in vitro observations. These findings suggest that KPT-330 attenuates RCC growth through inhibition of cell growth and induction of apoptosis in the RCC xenograft model, and that regulation of p21 localization plays a major role in the efficacy of KPT-330.

### KPT-330 resistant cells were sensitive to FDA approved therapeutics for RCC

**Figure 6:**
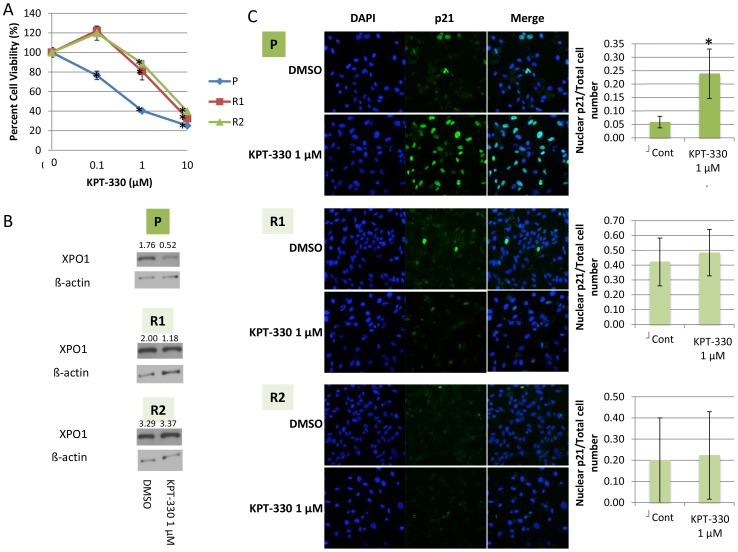
KPT-330 did not affect viability, XPO1 level, and p21 localization of KPT-330 resistant cells. KPT-330 resistant 786-O (R1 and R2) cells were established as described in the text, and these as well as the parental (P) cells were treated with KPT-330 at the indicated doses for 72 hours after which MTT assays were performed (A). R1, R2, and P cells were treated with KPT-330 for 24 hours then immunoblotting and immunofluorescence were performed. The number of cells in which p21 was predominantly in the nucleus were counted in three randomly selected fields and divided by the total cell number (Blue, nucleus (DAPI); Green, p21) (B). *P<0.05 compared to control. Error bars indicate standard deviation.

Due to the frequent occurrence of chemotherapy resistance in RCC patients treated with targeted therapeutics, it is critical to provide alternative therapeutic options for those who may develop resistance to XPO1 inhibition with the strategy proposed in this study. To evaluate the mechanism by which RCC cells become resistant to KPT-330, we focused on VHL-mut 786-O cells, since VHL mutation is seen in approximately 90% of RCC patients [18]. 786-O cells were initially incubated with a low (50 nM) concentration of KPT-330, and serially transferred to media with increasing concentrations of KPT-330 over a period of six months. Cells which started to grow at 1 µM KPT-330 were defined as “KPT-330 resistant”; from the parental 786-O cells (P), a total of two separate resistant cell lines were established (R1 and R2). As defined, R1 and R2 cells were less sensitive to KPT-330 than the P cells when treated with various doses of KPT-330 (Fig. 6A) with no attenuation of XPO1 (Fig. 6B). Interestingly, localization of p21 was unaffected in KPT-330 resistant cells (Fig. 6C), further supporting the importance of this protein in KPT-330 signaling.

**Figure 7:**
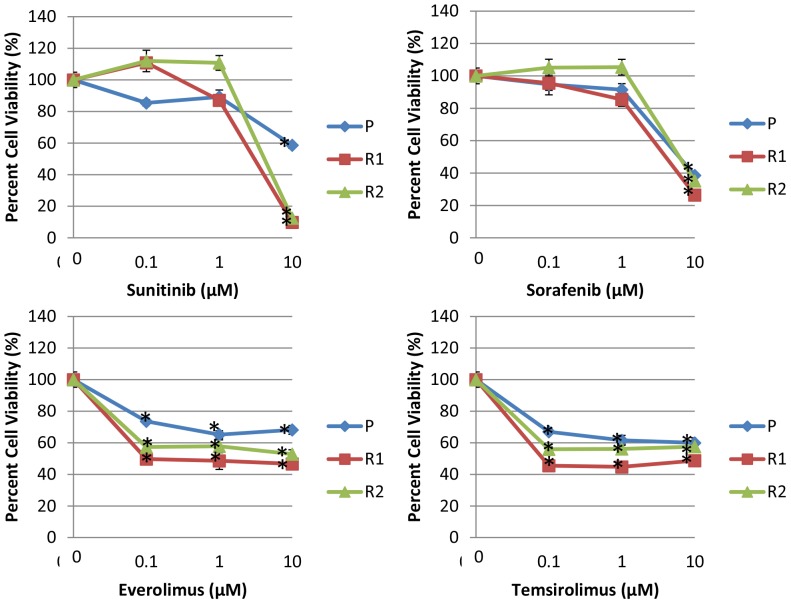
KPT-330 resistant cells were sensitive to FDA approved therapeutics for RCC. KPT-330 resistant 786-O (R1 and R2) and parental (P) cells were treated with KPT-330 at the indicated doses for 72 hours after which MTT assays were performed. *P<0.05 compared to control. Error bars indicate standard deviation.

To evaluate the potential for salvage therapies, survival of the resistant cells was assessed with conventional targeted agents. When KPT-330 resistant cells were treated with each of the currently approved for RCC kinase inhibitors (sunitinib and sorafenib) and mTOR inhibitors (everolimus and temsirolimus), R1 and R2 cells retained sensitivity similarly to P cells (Fig. 7), implying that the conventional targeted therapeutics could be utilized as second line therapy if patients develop resistance to KPT-330.

## Discussion

New therapeutics for advanced RCC have been approved by the FDA nearly every year since the advent of sorafenib, the first targeted RCC therapeutic. However, even with these drugs, extended PFS for those with metastatic RCC is only one to two years due to the development of drug resistance; the situation for patients with advanced disease is made more dismal by the fact that there are but a limited repertoire of available therapeutic targets (currently only kinases and mTOR) [4]. Thus it is critical to identify new targets for RCC which are distinct from those currently utilized. In light of earlier studies showing that XPO1 inhibitors have a therapeutic effect in RCC in vitro and in vivo [13], in this study we evaluated the efficacy and mechanism of KPT-330, the XPO1 inhibitor which is currently phase 1 trials, to determine potential clinical use of KPT-330 for advanced RCC. Indeed, outcomes of several ongoing Phase I/II clinical trials of KPT-330 (selinexor; clinicaltrials.gov) suggest that oral KPT-330 has clear anti-cancer activity with acceptable tolerability across multiple solid and hematological malignancies, yet there is scant data on this agent in RCC and no mechanistic information.

Cytosolic p21 is an indicator of poor prognosis in RCC, and it could conceivably be utilized as such a marker in other cancers, such as breast cancer, that are also characterized by overexpressed cytosolic p21 [10]. Both when incubated *in vitro* in several RCC cell lines and when orally administered *in vivo* in a human mouse RCC xenograft, KPT-330 increased nuclear p21 through specific inhibition of XPO1, resulting in a significant decrease of RCC cell viability through cell cycle arrest (and inhibition of proliferation in vivo) and induction of apoptosis. Surprisingly, there was minimal effect of KPT-330 on normal renal tubular epithelial cell viability or p21 localization in these cells, a finding which supports the likelihood of minimal adverse effects on renal (as well as other organ) function in patients who are administered this drug. When in the nucleus, p21 arrests the cell cycle by binding cyclin/CKD complexes at all phases, while when in the cytosol, p21 inhibits apoptosis by interacting with pro-apoptotic proteins including pro-caspase 3 and ASK [5]. Thus our findings suggest that nuclear p21 is involved in the mechanism by which KPT-330 affect RCC cells, a hypothesis supported by the fact that NHK cells whose p21 localization was not affected by KPT-330 were not as sensitive to KPT-330 as RCC cells and that KPT-330 resistant cells failed to localize p21 to the nucleus.

In many cancers, nuclear export of proteins is enhanced during disease progression and may in fact contribute to the process of drug resistance [19]. For example, higher levels of XPO1 expression are positively correlated with grade progression as well as increased proliferation rates in glioma [20], pancreatic cancer [21], and cervical cancer [22]. Based on our data, it is likely that nuclear export of p21 by XPO1 is more pronounced in RCC than in NHK cells as cytosolic p21 in the tumors is increased, especially in those RCC cases with poorer prognoses [9]. This observation (less effects by XPO1 inhibition in normal cells than their cancer cells) is consistent with other published studies with SINE showing that SINE target diseased cells while sparing normal cells [12]. Moreover, our findings that KPT-330 resistant cells retained sensitivity to all of the current FDA approved targeted therapeutics for advanced RCC (sunitinib, sorafenib, everolimus, and temsirolimus) suggests that patients who fail KPT-330 or for whom resistance develops, could still respond to these older drugs [4].

## Conclusions

The data shown here indicate that XPO1 inhibition by KPT-330 attenuates RCC viability through cell cycle arrest as well as induction of apoptosis, and that increased nuclear p21 by XPO1 inhibition plays a major role in the efficacy of KPT-330 in RCC. We show that KPT-330 potentiates the antitumor activity of sunitinib, resulting in complete inhibition of RCC tumor growth in vivo with this combination. Furthermore, our data support the likelihood that patients resistant to KPT-330 will respond to older targeted therapeutics. Given that advanced RCC has a poor prognosis even with targeted therapeutics, our work introduces KPT-330 as a novel therapeutic for RCC, which can rapidly be moved into the clinical realm to evaluate the efficacy by itself or combination treatment with other RCC therapeutics.

## Supporting Information

Figure S1 **KPT-330 induced apoptosis in RCC cells.** The RCC cells 786-O and ACHN were grown to ∼60% confluence. An Annexin V assay was performed after 24 hour incubation with DMSO or KPT-330 (1 µM) as described in Materials and Methods. The gating is shown.
(PPTX)
