# Supplementary figures and images for: Specific Inhibition of the Nuclear Exporter Exportin-1 Attenuates Kidney Cancer Growth
Source: PLoS One. 2014 Dec 2;9(12):e113867. doi: 10.1371/journal.pone.0113867 (PMC4252068; doi:10.1371/journal.pone.0113867)

## Slide 1
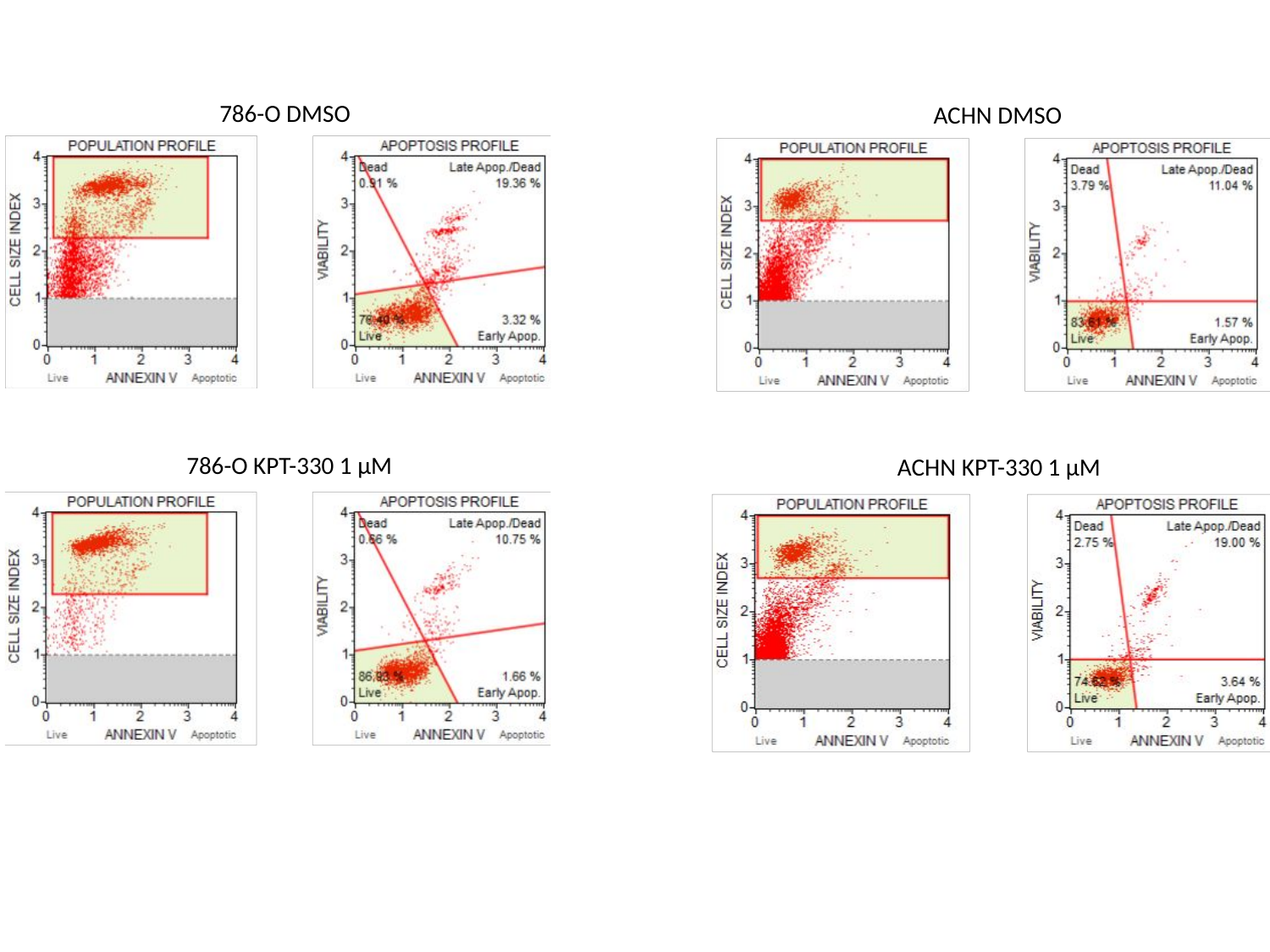

786-O DMSO
ACHN DMSO
786-O KPT-330 1 µM
ACHN KPT-330 1 µM

Supplement: Figure S1 — KPT-330 induced apoptosis in RCC cells. The RCC cells 786-O and ACHN were grown to ∼60% confluence. An Annexin V assay was performed after 24 hour incubation with DMSO or KPT-330 (1 µM) as described in Materials and Methods. The gating is shown. (PPTX) [file pone.0113867.s001.pptx]
